# The unresolved problem of beta-2 microglobulin amyloid deposits in the intervertebral discs of long-term dialysis patients

**DOI:** 10.1186/s13018-017-0697-6

**Published:** 2017-12-21

**Authors:** Tsung-Ting Tsai, Arun-Kumar Kaliya-Perumal, Chang-Chyi Jenq, Chi-Chien Niu, Natalie Yi-Ju Ho, Tung-Ying Lee, Po-Liang Lai

**Affiliations:** 1 Department of Orthopaedic Surgery, Spine Division, Bone and Joint Research Center, Chang Gung Memorial Hospital and Chang Gung University College of Medicine, Taoyuan, Taiwan; 2 Department of Orthopaedic Surgery, Melmaruvathur Adhiparasakthi Institute of Medical Sciences and Research, Affiliated to the Tamil Nadu Dr. MGR Medical University, Tamil Nadu, India; 3 Department of Nephrology, Chang Gung Memorial Hospital and Chang Gung University College of Medicine, Taoyuan, Taiwan; 4 Department of Medical Education, Chang Gung Memorial Hospital, Taoyuan, Taiwan

**Keywords:** Amyloid, Beta-2 microglobulin, Dialysis, Spondyloarthropathy, Congo red, Intervertebral disc

## Abstract

**Background:**

Dialysis-related destructive spondyloarthropathy caused by beta-2 microglobulin (β2M) amyloid deposits in intervertebral discs is a major burden for patients undergoing long-term dialysis. This study aimed to quantify the presence of β2M amyloid deposits in the intervertebral disc tissue of such patients and analyze whether there was a significant correlation between β2M accumulation and the duration of dialysis.

**Methods:**

Two groups of patients who had undergone surgery for degenerative spinal pathologies were selected: the dialysis group (*n* = 29) with long-term dialysis and the control group (*n* = 10) with no renal impairment. Tissue sections were prepared from specimens of intervertebral disc tissue obtained during spinal surgery and analyzed via histological staining, including immunohistochemistry (IHC) and Congo red.

**Results:**

There was a statistically significant multifold increase of β2M expression in the disc tissue of long-term dialysis patients when compared to non-dialysis patients, as shown by both IHC (0.019 ± 0.023 μm^2^ vs. 0.00020 ± 0.00033 μm^2^, respectively; *p* = 0.012) and Congo red staining (0.027 ± 0.041 μm^2^ vs. 9.240 × 10^−5^ ± 5.261 × 10^−5^ μm^2^, respectively; *p* = 0.047). We also note a moderate strength positive correlation between the duration of dialysis and positive IHC (*r* = 0.39; *p* = 0.015) and Congo-red staining (*r* = 0.42; *p* = 0.007).

**Conclusions:**

The problem of β2M amyloidosis in long-term dialysis patients remains unresolved even with predominant use of high-flux dialysis membranes. This highlights the insufficiency of current dialysis modalities to effectively filter β2M.

## Background

The incidence and prevalence of end-stage kidney disease (ESKD) has increased throughout the world [1–3], becoming a major public health problem with high economic and social costs [4, 5]. The number of patients receiving dialysis therapy has rapidly increased to an all-time high, especially in developing nations [5]. A large proportion of these patients are elderly and predisposed to increased risks for multiple adverse outcomes, such as hypotension, anemia, myopathy, and atrial fibrillation [6–9]. One of the major problems in such patients undergoing long-term hemodialysis is beta-2 microglobulin (β2M) amyloidosis [10]. The accumulation of β2M amyloid fibrils in the musculoskeletal system can lead to various degenerative conditions, depending on the musculoskeletal site of involvement [11, 12]. In the spine, such accumulation can damage the intervertebral discs and their adjacent endplates, leading to spinal instability and neurological compromise [13]. This destructive amyloid spondyloarthropathy is a major cause of disability in long-term dialysis patients.

There are two types of dialysis treatment, hemodialysis (HD) and peritoneal dialysis (PD), which are extensively practiced to manage ESKD. However, clinical outcomes may vary depending on different factors, such as age, comorbidities, and dialysis time. Studies demonstrate that PD offers equivalent or better survival than HD in the first year of treatment, especially in patients with advanced age and diabetes [14]. In addition, there are no reported differences in the occurrence of β2M amyloidosis and musculoskeletal complications between patients treated with HD and PD [11]. However, β2M levels are known to be influenced by the membrane used for HD treatment. For example, the levels of β2M are said to be significantly reduced in patients treated with high-flux HD, which uses polysulfone membranes, when compared to patients treated with conventional dialysis using cellulose membranes [15].

Recently, high-flux HD is widely performed to improve the quality and efficacy of dialysis treatment. With the advent of this technique, occurrence of destructive amyloid spondyloarthropathy should be less likely, though it has to be studied. To address this question, we quantified the presence of β2M amyloid deposits in intervertebral discs among long-term dialysis patients who had undergone HD or PD and analyzed whether there was a significant correlation between β2M accumulation and the duration of dialysis.

## Methods

Retrospectively, we reviewed records of patients with ESKD who were on long-term dialysis therapy, but also underwent spinal surgery for single or multilevel degeneration with or without spinal instability. We shortlisted the patients for whom formalin-fixed paraffin wax embedded disc tissue samples were available for further analysis, resulting in a sample size containing a dialysis group of 29 samples. A small control group of 10 samples with similar demographic characteristics (e.g., age, body-mass index, surgical level and location), but without any renal disease, were selected for comparison (i.e., the non-dialysis group). This selection method was non-randomized as only those patients whose disc tissue samples were available for further analysis were chosen.

We tabulated several basic parameters, including age, gender, underlying disease, and the severity of disc degeneration. Chronic kidney disease, mineral and bone disorder-related parameters, such as serum levels of alkaline phosphatase, calcium, and phosphate, were considered. We also included several dialysis-related parameters, such as type, duration, and adequacy (*Kt*/*V*; in which *K* = the dialyzer clearance, *t* = time, and *V* = urea distribution volume) of the dialysis treatment. The severity of disc degeneration at the surgical level among patients of both groups was graded according to Pfirrmann’s classification using magnetic resonance imaging (MRI) images [16]. We acquired and prepared slides of the formalin-fixed paraffin wax embedded disc tissue samples from the Tissue Bank. The disc specimens were fixed in neutral buffered formalin, embedded into paraffin wax blocks, and stored at room temperature at the Tissue Bank with no storage time limit. These unstained slides were deparaffinized in xylene, rehydrated through a series of graded alcohol washes, and soaked in distilled water before staining. Congo red and immunohistochemistry (IHC) staining were then performed on all acquired samples.

### Congo red staining

After sample preparation, the sections from all 39 samples were stained with Mayer’s Hematoxylin Solution (MHS1-100ML, Sigma-Aldrich, St. Louis, MO) for 10 min and thoroughly washed for 5 min, after which they were immediately soaked with alkaline sodium chloride solution for 20 min, followed by alkaline Congo red solution for 20 min (HT60 kit, Sigma-Aldrich). The sections were dehydrated with a series of graded alcohol washes and then mounted for evaluation under normal and polarized light microscopy.

### Immunohistochemistry staining

After sample preparation, the 39 sections were washed twice in PBS (Sigma-Aldrich) for 5 min. The sections were blocked with a solution of 10% normal serum (FBS; Gibco, Grand Island, NY) and 1% BSA (Sigma-Aldrich) in PBS for 3 h and then incubated with anti-β2M antibody (ab195531, Abcam, Cambridge, MA) at 1:400 dilution for 30 min. We added 3,3′-diaminobenzidine substrate (Abcam) and incubated the sectioned samples for 5 min, followed by thorough washing with tap water for another 5 min, and finally staining with Mayer’s Hematoxylin Solution for 10 min. The sections were dehydrated with a series of graded alcohol washes and then mounted for evaluation under light microscopy.

### Protein extraction and western blotting

Intervertebral disc tissue samples were obtained with written informed consent from two participants: one was a non-dialysis patient who underwent an L5-S1 discectomy, and the other was a patient who had received HD for 13 months before undergoing an L4-L5 discectomy. After washing the surgically harvested tissues with PBS twice, the tissues were mashed with a surgical blade. Next, 500 μl of T-PER Tissue Protein Extraction Reagent (78510, Thermo Fisher Scientific, Rockford, IL) was added to 0.05 g of the tissue, after which an ultrasonic homogenizer (Q700; QSonica, LLC, Newtown, CT) was used to breakdown the samples. The protein material was then rested on ice for 30 min and then centrifuged for 15 min at 13,000 rpm at 4 °C. The resulting supernatant was collected. The protein sample was then separated using SDS-PAGE, transferred onto a polyvinylidene difluoride membrane, and soaked with blocking reagent for 3 min. This was followed by overnight incubation in antibody dilution buffer containing the primary anti-β2M antibody (1:2000; Abcam), as well as anti-beta actin monoclonal antibody (1:5000; Proteintech, Rosemont, IL) as an internal control. After washing away the unbound antibodies with TTBS (1× TBS buffer and 0.1% Tween 20; Sigma-Aldrich) three times for 5 min each, rabbit anti-mouse secondary antibody (1:10,000) was incubated for 2 h, followed by washing with TTBS again, and visualized by Pierce Enhanced Chemiluminescence Western Blotting Substrate (Thermo Fisher Scientific).

### Statistics

Quantitative analysis of histological staining was performed using Image J (National Institute of Health, Bethesda, MD) to estimate the proportion of positively stained area in the sectioned intervertebral disc tissue samples. Measurements were done for both IHC and Congo red stained sections among both groups, which were tabulated for comparison. A correlation analysis was performed to evaluate the strength of association between the duration of dialysis and the positively stained area by either of the staining methods. The percentage of the positively stained area over the total area was calculated.

Statistical analyses were performed using IBM SPSS Statistics for Windows, Version 19.0 (IBM Corp., Armonk, NY). For data analysis, the Student’s *t* test was used for continuous variables and Fisher’s exact test for categorical variables. A *p* value of < 0.05 was considered statistically significant.

## Results

Table 1 summarizes the demographic data among the 39 samples that were selected for the study. The dialysis group had a mean age of 62.4 ± 8 years, and the control non-dialysis group a mean age of 59.4 ± 6.5 years. The numbers of patients with single- or multilevel disc degeneration in both groups were statistically similar. Predominantly, surgeries were done for patients with lumbar degeneration, except for 4 samples among the dialysis group for whom the surgery was done for single-level cervical degeneration. However, this small number did not result in any statistical difference between the groups. The number of patients with associated comorbid conditions, including diabetes *mellitus* and hypertension, were similar in both groups. There was no significant difference between the dialysis and non-dialysis patients in terms of their serum-based alkaline phosphatase, calcium, and phosphate levels. For the dialysis group, there were 12 grade III, 4 grade IV, and 5 grade V discs. For the non-dialysis group, there were 7 grade III, 2 grade IV, and 1 grade V discs. The severity of disc degeneration also did not show any statistical difference between the groups. Therefore, we considered the groups to be appropriately matched.

**Table 1:** Comparison of demographic data between the dialysis and non-dialysis groups

Parameters	Dialysis group	Non-dialysis group	*p* value
	*n* = 29	*n* = 10	
Age [mean ± SD (range)]	62.4 ± 8 (44–75)	59.4 ± 6.5 (48–67)	0.28
BMI	23.86 ± 3.77	23.91 ± 3.94	0.97
Level			
Single-level	14	6	0.72
Multi-level	15	4	
Location			
Cervical	4	0	0.55
Lumbar	25	10	
Comorbidity			
Diabetes *mellitus*	12	2	0.27
Hypertension	26	6	0.06
Serum level			
Serum ALP	142.2 ± 73.09	102.4 ± 35.8	0.11
Serum Ca	10.1 ± 0.86	9.7 ± 0.56	0.18
Serum P	4.65 ± 2.01	3.86 ± 0.14	0.23
Dialysis duration (months)	82.5 ± 44.3	–	–
Dialysis adequacy (Kt/V)	1.58 ± 0.19	–	–

*SD* standard deviation, *BMI* body mass index, *ALP* alkaline phosphatase, *Ca* calcium, *P* phosphorous

For those in the dialysis group, dialysis was done for a mean duration of 82.5 ± 44.3 months, ranging from 17.08 to 194.4 months (Table 1). There were 22 samples on high-flux HD and 7 samples on PD. The mean time interval between specimen collection and histological examination was 23.83 ± 19.5 months (range, 0–76 months) in the dialysis group. However, the time interval was only a few months for those in the non-dialysis group.

Histology examination of the sections following Congo red staining under polarized light showed areas of apple-green birefringence that indicated amyloid deposits. The mean positively stained area among HD patients of the dialysis group was 0.025 ± 0.045 μm^2^, while for PD patients it was 0.034 ± 0.028 μm^2^. The consolidated measurement for the dialysis group (0.027 ± 0.041 μm^2^) proved to be significantly higher than that of the non-dialysis group (9.240 × 10^−5^ ± 5.261 × 10^−5^ μm^2^; *p* = 0.047). Similarly, following IHC staining, the HD and PD group were immunopositive, measuring 0.021 ± 0.023 μm^2^ and 0.013 ± 0.023 μm^2^, respectively. Their consolidated measurement (0.019 ± 0.023 μm^2^) was also significantly higher than that of the non-dialysis group (0.00020 ± 0.00033 μm^2^; *p* = 0.012). However, there was no significant difference in the positively stained areas between the HD and PD patients using either IHC or Congo red staining (Table 2).

**Table 2:** Comparison of positively stained areas between the dialysis and non-dialysis groups using IHC and Congo red staining

Staining	Dialysis group (μm^2^)		Non-dialysis group (μm^2^)	*p* value^a^
IHC	HD	0.021 ± 0.023	0.00020 ± 0.00033	0.012
	PD	0.013 ± 0.023		
	*p* value^b^	0.42		
Congo red	HD	0.025 ± 0.045	0.0000924 ± 0.00005261	0.047
	PD	0.034 ± 0.028		
	*p* value^b^	0.63		

*IHC* Immunohistochemistry staining, *HD* hemodialysis, *PD* peritoneal dialysis

^a^Analyzed between dialysis and non-dialysis groups

^b^Analyzed between HD and PD

**Fig. 1:**
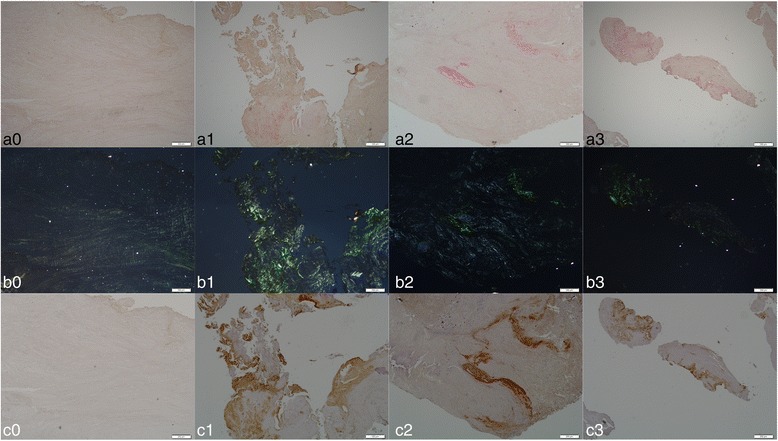
Representative histological images of intervertebral disc sections from non-dialysis (0) and dialysis patients (1–3). **a** Congo red staining under normal light and amyloid deposition indicated in dark red. **b** Congo red staining under polarized light and amyloid deposition indicated in apple green. **c** IHC staining and β2M expression indicated in dark brown. Magnification: ×40

Assessment of correlation between the duration of dialysis and the positively stained area as measured by either of the staining methods revealed a moderate strength positive correlation between the two (IHC staining, *r* = 0.39, *p* = 0.015; Congo red staining *r* = 0.42, *p* = 0.007). Quantitative results showed the percentage of IHC-stained area over the total tissue area was 2.30% for the dialysis group and 0.01% for the non-dialysis group. The percentage of Congo red stained area over the total area of the sectioned samples was 1.49% for the dialysis group and 0.009% for the non-dialysis group, which suggested that the expression of β2M amyloid deposits was 230-times and 165-times higher in the disc tissue of patients who had undergone dialysis therapy than in the tissue of non-dialysis patients, based on IHC and Congo red staining, respectively (Fig. 1).

**Fig. 2:**
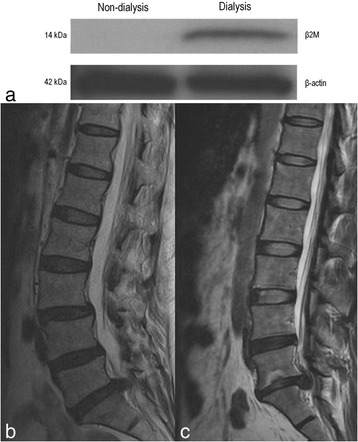
Western blot analysis of the intervertebral disc tissues of one non-dialysis and one dialysis patient. **a** Western blot analysis of β2M using beta-actin as a loading control. **b** Sagittal cut MRI image from the non-dialysis patient showed the right L5/S1 herniated nucleus pulposus with grade III degeneration. **c** Sagittal cut MRI image from the dialysis patient showed left L5/S1 extrusion type herniated nucleus pulposus with Grade IV degeneration

Western blot analysis indicated that β2M protein was present in the intervertebral disc tissue obtained from the patient who had received 13 months of HD therapy, but was absent in the disc tissue obtained from the non-dialysis patient (Fig. 2). Quantifying the band intensity using Image J, the ratio of β2M to β-actin for the dialysis patient was 147.77 times higher than that of the non-dialysis patient, which confirmed a significantly higher expression of β2M protein in the intervertebral disc tissue of the patient who underwent HD treatment when compared to the disc of the patient without dialysis treatment.

## Discussion

β2M is constantly produced in the body and eliminated exclusively by the kidneys, mostly by glomerular filtration, after which 99.9% of the excreted proteins are reabsorbed in the renal tubules, where they are catabolized [17]. When glomerular filtration fails, increased serum levels of β2M are observed. This mechanism is the same for the accumulation of β2M in dialysis patients, as there is a bio-incompatibility that prevents the dialysis membranes from efficiently filtering β2M. Studies have shown that β2M levels significantly increase after conventional HD using cellulose acetate membranes [15].

However, the science of dialysis is constantly evolving, and the current standard method is different from the past conventional techniques and membranes. With the advent of high-flux dialysis, β2M levels are significantly decreased after dialysis [13]; hence, destructive amyloid spondyloarthopathy should be less likely to occur. However, even though a type of high-flux dialysis membrane was used in the majority of our patients, we still found β2M accumulation in their intervertebral discs. This signifies that even with recent advances in dialysis, β2M amyloidosis remains an unresolved issue. Moreover, conventional dialysis methods are still used in most developing countries [18], which further contributes to the prevalence of β2M amyloidosis.

Literature suggests that dialysis itself is an inflammatory stimulus, which can induce cytokine production (e.g., Interlukin-1, Interlukin-6, and tumor necrosis factor-α) and activate complement systems. Subsequently, the released cytokines stimulate the synthesis of β2M and its release by macrophages [19]. β2M is often highly expressed in articular cartilage of various joints and the synovial fluid, leading to compromised chondrocyte function that results in osteoarthritis [20, 21]. Apart from musculoskeletal involvement, β2M amyloid deposits can also be found in arterioles, venules, and muscularis propria of visceral organs, like those of the gastrointestinal tract and heart [22, 23].

Intervertebral discs, facet joints, and ligamentum flavum are the most susceptible sites for β2M accumulation in the vertebral column [24, 25]. This can lead to destructive spondyloarthropathy (DSA) and consequential disability [26]. DSA is increasingly recognized as a complication of long-term HD [24]. Clinical symptoms depend on the sites affected by β2M amyloid deposition. Most patients are asymptomatic; however, some might complain about localized back pain, which is often accompanied by arm pain or numbness [15, 27]. The radiological features include narrowing of disc space and the destruction of adjacent vertebral endplates [15]. This may precede segmental instability or neurological compromise [28, 29]. Our study included patients predominantly with lumbar disc degeneration and a few patients with cervical disc involvement. This non-random selection may not represent the demographics of the entire population. However, cervical discs are said to be more commonly affected, followed by the lumbar and upper thoracic discs, and then the middle and lower thoracic discs [24].

Disc degeneration can be graded according to Pfirrmann’s classification system [16]. However, we had 8 samples in the dialysis group who did not fit into any grade. This was due to the difficulty in deciding whether the radiological changes were caused by dialysis-related spondyloarthropathy or spondylodiscitis [27]. Usually, DRS presents as low signal intensity in the MRI of both T1 and T2 weighted images, unlike spondylodiscitis which presents as low signal intensity on T1 and high signal intensity on T2 weighted images [15, 27]. However, unusual signal intensity changes as we observed in a few of our cases can also be present; but post-operative culture of the disc material revealed no microorganisms suggestive of infection. Therefore, we considered these patients to have dialysis-related spondyloarthropathy.

Histological examination is the gold standard for diagnosing β2M amyloidosis. Our histological study included Congo red and IHC staining of disc material sections. Positive Congo red staining, appearing as distinct birefringence under polarized light, and immunostaining of amyloid deposits with a labeled anti-β2M antibody are considered pathognomonic for the presence of β2M [30]. We also performed western blot analysis, but only for one patient from the dialysis group and one from the non-dialysis group. Our results suggest a multifold increase in β2M expression in the disc tissue of long-term dialysis patients. There was also a moderate strength positive correlation between the duration of dialysis and positive IHC and Congo red staining. These findings from our pilot analysis provide important insights into our understanding of β2M amyloidosis and highlight the need for dialysis membranes that can more effectively filter β2M molecules to help prevent potential long-term complications [10, 31].

This study was a cross-sectional prevalence measure with certain limitations. Considering the sample size, this study may be underpowered to draw potential conclusions. Also, not all patients had definite radiological evidence of DRS before surgery. However, β2M deposition was noted among all dialysis group patients. Further empirical evidence could be collected by performing western blot analysis for all the patients in the study.

## Conclusions

We analyzed the presence of β2M amyloid deposits in the intervertebral discs of long-term dialysis patients using Congo red, IHC staining and western blot analysis of surgically harvested tissue. Our results showed a statistically significant multifold increase of β2M expression in the disc tissues of long-term dialysis patients when compared to non-dialysis patients. We also inferred a moderate strength positive correlation between the duration of dialysis and positive IHC and Congo red staining, representing the presence of β2M amyloids. Our findings highlight the issue of β2M amyloidosis still prevailing among long-term dialysis patients even with the advent of high-flux dialysis, therefore warranting further study.

## Abbreviations

ALP: Alkaline phosphatase
BMI: Body mass index
Ca: Calcium
DSA: Destructive spondyloarthropathy
ESKD: End-stage kidney disease
HD: Hemodialysis
IHC: Immunohistochemistry
MRI: Magnetic resonance imaging
P: Phosphorous
PD: Peritoneal dialysis
SD: Standard deviation
β2M: Beta-2 microglobulin
